# Physical Enrichment Triggers Brain Plasticity and Influences Blood Plasma Circulating miRNA in Rainbow Trout (*Oncorhynchus mykiss*)

**DOI:** 10.3390/biology11081093

**Published:** 2022-07-22

**Authors:** Emilie Cardona, Valentin Brunet, Elodie Baranek, Léo Milhade, Sandrine Skiba-Cassy, Julien Bobe, Ludovic Calandreau, Jérôme Roy, Violaine Colson

**Affiliations:** 1 INRAE, INRAE, Université de Pau & Pays Adour, NUMEA, 64310 Saint-Pée-sur-Nivelle, France; emilie.cardona@inrae.fr (E.C.); elodie.baranek@inrae.fr (E.B.); sandrine.skiba@inrae.fr (S.S.-C.); 2 INRAE, LPGP, 35000 Rennes, France; valentin.brunet@inrae.fr (V.B.); julien.bobe@inrae.fr (J.B.); 3 IRISA, INRIA, CNRS, Université de Rennes 1, 35000 Rennes, France; leo.milhade@gmail.com; 4 CNRS, IFCE, INRAE, Université de Tours, PRC, 37380 Nouzilly, France; ludovic.calandreau@inrae.fr

**Keywords:** environmental enrichment, behavioral plasticity, transcriptomic, circulating miRNA, cerebral function

## Abstract

**Simple Summary:**

Overall, this study has reported that environmental enrichment significantly displayed a series of differentially expressed genes and pathways related to cerebral activity, neural plasticity (neurotrophic markers), neurogenesis, and synaptogenesis, essentially in telencephalon, which may underpin the beneficial effects of a complex environment on rainbow trout’s adaptive behaviors. In addition, environmental enrichment significantly influenced circulating miRNAs (c-miRNAs) profiles of plasma in rainbow trout, which reveals the high potential of c-miRNAs, as physiologically relevant biomarker candidates of fish behavioral plasticity.

**Abstract:**

Physical enrichment is known to improve living conditions of fish held in farming systems and has been shown to promote behavioral plasticity in captive fish. However, the brain’s regulatory-mechanism systems underlying its behavioral effects remain poorly studied. The present study investigated the impact of a three-month exposure to an enriched environment (EE vs. barren environment, BE) on the modulation of brain function in rainbow trout (*Oncorhynchus mykiss*) juveniles. Using high-throughput RT-qPCR, we assessed mRNA genes related to brain function in several areas of the trout brain. These included markers of cerebral activity and plasticity, neurogenesis, synaptogenesis, or selected neurotransmitters pathways (dopamine, glutamate, GABA, and serotonin). Overall, the fish from EE displayed a series of differentially expressed genes (neurotrophic, neurogenesis, and synaptogenesis markers) essentially localized in the telencephalon, which could underpin the beneficial effects of complexifying the environment on fish brain plasticity. In addition, EE significantly affected blood plasma c-miRNA signatures, as revealed by the upregulation of four c-miRNAs (miR-200b/c-3p, miR-203a-3p, miR-205-1a-5p, miR-218a-5p) in fish blood plasma after 185 days of EE exposure. Overall, we concluded that complexifying the environment through the addition of physical structures that stimulate and encourage fish to explore promotes the trout’s brain function in farming conditions.

## 1. Introduction

The environment of an organism can convey a powerful influence over its biology and physiology. Since as early as the late 1940s, with Hebb’s first highlighted ‘enriched environment (EE)’ as an experimental concept [1], it has become well-known that environmental stimulation promotes a wide range of effects on an animal’s behavior [2]. An EE is defined as “a modification of the environment of captive animals, thereby increasing the animal’s behavioral possibilities and leading to improvements of their biological function” [3]. Although results vary depending on the outcome variables assessed, the addition of different structures to the habitat (e.g., physical enrichment) and types of environmental enrichments (e.g., sensorial, occupational, social, and dietary enrichment), as reviewed in fish by Arechavala-Lopez and collaborators [4], confers more environmental complexity and deeply provides behavioral plasticity by modifying an individual’s environmental perception. Indeed, the surrounding environments that animals inhabit shape their behavioral phenotypes, physiological status, and molecular processes.

In fish, a growing body of studies describes the beneficial effects of EE but to a lesser extent than in mammals (for reviews, see Corcoran [5] and Näslund and Johnsson [6]). However, EE has been shown to improve fish cognitive abilities [7], to reduce aggressive behavior [8], and to circulate stress hormones [9]. However, EE is not always beneficial. While some studies showed improved cognition and brain growth in fish kept in structured environments, other studies found no effect [10,11,12] from physical enrichment. Moreover, a recent review by Jones and collaborators [13] highlighted the importance of the type of physical environment (dimensions, ecological rationale, timing of enrichment, amount, inputs, lighting, and social environment), which could explain the different results obtained in these studied.

Recently, we highlighted important behavioral differences between rainbow trout (*Oncorhynchus mykiss*) juveniles reared in EE with physical enrichments (PVC pipes, plastic plants, and white stones) for three months and those held in barren environment (BE) [14]. When observed in their rearing tanks, enriched fish displayed more shoal cohesion and fewer agonistic and/or stereotypic behaviors. When subjected to social isolation in a novel environment, they explored more and displayed fewer fear-related behaviors. When exposed to a novel object, enriched fish were bolder and less neophobic, spending more time close to the object, without exhibiting anxiety-like behaviors. This behavioral plasticity triggered by EE is closely linked to the individual’s perception of its surroundings (threatening, safe, etc.), which is an elementary process involved in cognition [15].

In sharp contrast, the relation between observed fish behaviors triggered by EE and the brain, which underlies these behaviors, remains largely unknown. Ebbesson and Braithwaite [16] synthetized the impacts of the environment, whether natural or in aquaculture, on fish neural plasticity and cognition. Most studies of EE-induced brain changes have described effects on brain size [17,18,19] and, generally, EE results in faster rates of neural proliferation [7,20,21]. However, the neural regulatory mechanisms underpinning the specific effects of EE on cognition have rarely been studied in fish, and even more rarely in salmonids. Among these species, trout is the main continental fish species produced in Europe (European Commission, 2020), so it deserves more attention, especially for finding strategies to alleviate welfare issues caused by low-stimulating environments often encountered in farming systems. Understanding the neural mechanisms underlying the behavioral effects of EE, which we previously reported in trout [14], would highlight the brain-plasticity mechanisms involved and provide new insight into the benefits of this practice in farmed fish.

Several studies have revealed a specific role of small non-coding RNAs (c-miRNAs, approximately 22 nucleotides in length) in a wide variety of biological processes in mammals, including neuronal plasticity and other brain functions [22,23], and these would play a potential role in cognitive functions [24]. Furthermore, previous studies reported that the expression of c-miRNAs is associated with behavioral responses to drugs [25,26], behavioral plasticity in honey bees [27], and agonistic behavior in crab [28]. Numerous studies in humans have shown that c-miRNAs are naturally secreted from cells into body fluids, such as serum, blood plasma, saliva, urine, milk, amniotic fluid, colostrum, bronchial lavage, cerebrospinal fluid, peritoneal fluid, and pleural fluid [29], where they remain highly stable [30]. This confers a great potential to c-miRNAs as prognostic biomarkers. Recently, the high potential of c-miRNAs was highlighted as a physiologically relevant biomarker candidate for the phenotyping of reproductive and metabolic states in trout [31]. However, no research in fish has currently evaluated the association of c-miRNAs with cognitive and behavioral responses, despite the potential interest of these c-miRNAs as biomarker candidates of cognition-related behavioral plasticity, as revealed by analyses of numerous genes’ expressions.

The present study, complementing our previous work [14], aims to investigate the molecular mechanisms at the cerebral level underlying the EE’s beneficial effects in trout reared in an aquaculture system. In order to reveal the brain’s regulatory pathways and networks involved in the environmental effects on fish cognition, we used a high-throughput microfluidics quantitative polymerase chain reaction (qPCR) system (Biomark microfluidic system; Fluidigm, San Francisco, USA) to analyze numerous genes’ expressions in specific areas of the trout brain (forebrain, hypothalamus, and hindbrain). These selected genes include markers of cerebral activity, neural plasticity (neurotrophic markers), neurogenesis, synaptogenesis, and selected neurotransmitters pathways. In addition, we aim to explore the potential of selected c-miRNAs in a biological fluid (plasma) of fish as non-invasive biomarkers of the behavioral plasticity through the cognitive processes observed in our EE study.

## 2. Materials and Methods

### 2.1. Animal Handling

The experiment was conducted following the Guidelines of the National Legislation on Animal Care of the French Ministry of Research (Decree No 2013–118, February 2013) and in accordance with EU legal frameworks related to the protection of animals used for scientific purposes (i.e., Directive 2010/63/EU). The scientists in charge of the experiments received training and personal authorization. The experiment was conducted at the INRAE Physiology and Genomic Laboratory (LPGP) experimental facilities (permit number C35-238-6, Rennes, France), delivered by French veterinary services, and approved by the ethics committee for the animal experimentation of Rennes and the French minister of national education, research, and innovation, under the authorization number APAFIS#28962- 131 2021011323275224.

### 2.2. Fish and Experimental Design

The experimental design is already published [14]. In this study, we used triploid female trout (*Oncorhynchus mykiss*), the most commonly used in research experiments because they are the ones sold in the market. Fish were originated from eggs fertilized at experimental facilities (INRAE-PEIMA, permit number B29-277-02, Sizun, France). Eggs originated from two batches: eggs from batch 1 used for c-miRNAs analyses were fertilized on 7 November 2019, while eggs from batch 2 used for qPCR analyses were fertilized on 18 June 2020. Trout fry were transferred to INRAE LPGP (Rennes, France) at 53 and 99 days post fertilization (dpf) for small RNA and qPCR analyses, respectively. They were split into two experimental groups, submitted to either environmental enrichment (EE) or barren environment (BE) and kept under these conditions until 238 dpf for batch 1 and 189 dpf for batch 2. The EE consisted of physical enrichments and was composed of PVC pipes, plastic plants (grapes of leaves), and white stones, representing a complete panel of the different structures tested in the literature reviewed by Näslund and Johnsson [6]. For each stage, fish mean weight, tank size, number of individuals per tank, number of tanks per treatment, and number and types of physical enrichments, as well as estimated floor coverage (%), are given in Table 1 (batch 1 for c-miRNA analysis) and Table 2 (batch 2 for molecular analysis).

**Figure 1:**
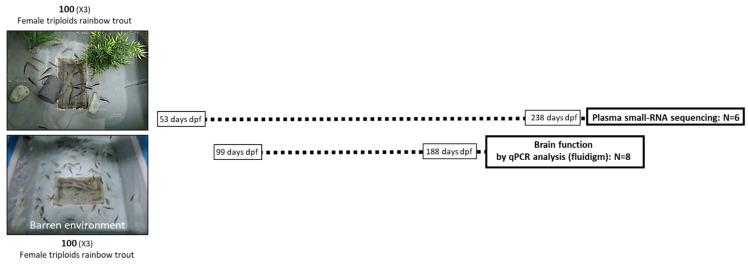
Scheme of experimental procedure.

Fish load was always lower than 25 kg/m^3^. Water temperature was maintained at 12 +/− 0.2 °C, the artificial photoperiod was 12:12 h, and the water quality was regularly checked (NH_4_^+^, NO_2_-159, NO_3_-160). All tanks were supplied by circulating and recycled water. Fish were fed daily with extruded pellets (39% proteins and 24% lipids, Le Gouessant, France), the diameter and the quantity being regularly adapted according to fish mean weight. The experimental protocol is summarized in Figure 1.

Fish rearing, fluid sampling, and biological samples used for RNA-seq and RT-qPCR analysis are described.

### 2.3. Sampling Protocol

For qPCR analyses, eight fish per treatment were euthanized using a lethal dose of tricaine methane sulphonate (200 mg/L; PHARMAQ, Hampshire, UK) after three months of being reared in either environmental enrichment (EE) or barren environment (BE). Subsequently, the fish brain was rapidly dissected, and the forebrain, hindbrain, and hypothalamus were extracted and frozen at −80 °C until qPCR analyses, as previously described [32].

For small RNA analyses, after a six-month captive period (in enriched or barren tanks), five fish (other cohort) from EE treatment and six fish from BE treatment were euthanized. Blood (~0.400 mL) was sampled from caudal sinuses into EDTA-coated syringes, and samples were stored on ice. After sampling, blood cells and plasma were separated by centrifugation (10 min at 3000 rpm). Plasma was collected and frozen at −20 °C until small RNA analyses.

In this study, the fish used for RNA-seq analyses were from batch 1, with fish that were never subjected to behavioral tests. Fish used for RT-qPCR analyses were also not subjected to behavioral tests prior to tissue collection but originated from the same batch 2 as the fish with behavioral phenotypes that were published in our previous paper [14].

### 2.4. Gene Expression Measurement by Real-Time Quantitative PCR

For 8 fish per treatment (EE and BE), total RNA was extracted from the brain divided in three areas (forebrain, hindbrain, and hypothalamus) using the TRIzol reagent method (Invitrogen, Carlsbad, CA, USA) with Precellys (Bertin technologies, Montigny le Bretonneux, France) in accordance with the instructions of the manufacturer, as previously described [33]. Total RNA (2 μg) was used for cDNA synthesis. The Super-Script III RNAse H-Reverse transcriptase kit (Invitrogen) was used with random primers (Promega, Charbonniéres, France) to synthesize cDNA. High throughput real-time quantitative PCR (RT-qPCR) was performed using the Biomark microfluidic system from Fluidigm (Fluidigm, San Francisco, CA, USA), in which each gene combination was quantified using a 96.96 Dynamic Array™ IFCs (BMK-M-96.96, Fluidigm) as previously published [34]. Pre-amplification of the samples, chip loading, and RT-qPCR were performed in accordance with the protocol of the manufacturer. The results were analyzed using the Fluidigm PCR analysis software v.4.1.3. Firstly, 6.5 ng of each cDNA were initially pre-amplified (10 min 95 °C activation and 14 PCR cycles at 15 s 95 °C for 4 min, 60 °C) with PreAmp Master Mix (100-5581, Fluidigm) with a pool containing all the primers (200 nM), excluding the 16S rRNA primer sets. Pre-amplified samples were diluted to 1/5 after an exonuclease treatment (M02935, NEB, Biolabs, Ipswich, MA, USA). In order to prepare samples for loading into the IFC, a mix was prepared consisting of 440 μL 2× TaqMan Master Mix (Applied Biosystem 4369016, Thermofischer Scientific, Maryland, USA), 44 μL 20× DNA Binding Dye Sample Loading Reagent (100-7609, Fluidigm), and 44 µL 20× EvaGreen dye (31000, Biotium, San Francisco, CA, USA), plus 132 μL TE buffer. A volume of 6 μL of the mix was dispensed to each well of a 96-well assay plate. Two microliters of pre-amplified and diluted cDNA sample were added to each well, and the plate was briefly vortexed and centrifuged.

For the assays, 5 μL of each Assay (5 μM each primer in primer-mix (2× assay loading reagent, 100-7611, Fluidigm) with Tris EDTA) were dispensed to each Detector Inlet of the 96.96 IFC. Following priming of the IFC in the IFC Controller HX, 5 μL of the cDNA sample + reagent mix and 5 μL of Assay were dispensed to each sample inlet of the 96.96 IFC. After loading the assays and samples into the IFC, it was transferred to the BioMark, and PCR was performed using the following thermal protocol: thermal mix of 50 °C, 2 min; 70 °C, 30 min; 25 °C, 10 min; hot start at 50 °C, 2 min; 95 °C, 10 min PCR Cycle of 35 cycles of (95 °C, 15 s; 60 °C, 60 s), and melting analysis (60 °C, 30 s; 95 °C, 1 °C/3 s). Results were analyzed using the Fluidigm real-time PCR analysis software v.4.1.3. Data were extrapolated from standard curves and normalized to the validated housekeeping gene. Compared to *18 s* and *actin*, the elongation factor 1α gene (*eef1α*) showed the most stable expression between brain areas and tissues and was chosen as the reference gene. Relative expression of the target genes was determined by the ΔΔCT method [35,36,37]. Mean ± standard error of mean values for each group are expressed in fold changes relative to the BE treatment for all genes. Primer sequences used to amplify all paralogs genes and accession numbers of the primers are presented in Table 1. All gene sequences of trout used were identified by in silico analysis by Genomicus software v.100.01 (www.genomicus.biologie.ens.fr; accessed on 1 November 2020) and Ensembl (http://www.ensembl.org, Ensembl Release 102; accessed on November 2020, trout genome available) and queried against mammals or fish genome using the BLAST tool in Ensembl and in NCBI (https://blast.ncbi.nlm.nih.gov/Blast.cgi, accessed on 1 November 2020) to confirm the gene identification (Table 3).

### 2.5. Primer Design

The primer sequences used to amplify all genes and accession numbers of the primers are presented in Table 3. For gene targets that had not previously been validated, primers were tested on a pool of cDNA, and amplified products were systematically sequenced.

PCR primers (Table 3) for the target regions were designed for each gene using Primer3 software [38]. To optimize annealing temperature for the respective gene, PCR was performed with specific primer pairs, and analysis of PCR products was done by electrophoresis on agarose gel (1.5%) [39].

### 2.6. RNA Extraction, Pre-Processing, and Illumina Small RNA Deep Sequencing for Plasma Samples

Plasma samples were homogenized in TRIzol reagent (Trizol BD) at a ratio of 400 µL of fluid per milliliter of reagent (N = 5 for EE, N = 6 for BE). Total RNA was extracted in accordance with the instructions of the manufacturer. Except for the isopropanol step, glycogen was added to each sample to facilitate visualization of precipitated RNA. The quantity of miRNA was assessed using qubit microRNA assay kit (ThermoFisher, Maryland, USA), which allows fast and accurate quantification of c-miRNA using the Qubit Fluorometer. Libraries from RNA were constructed using the NEXTflex small RNA ki v3 (Bioo Scientific, Saint Marcel, France). Starting from 1 µg of total RNA, an adapter was ligated on the 3’ end of the RNAs. A second adapter was ligated to the 5’ end. Ligated RNAs were subjected to reverse transcription using M-MuLv transcriptase and a trout primer complementary to the 3’ adapter. PCR amplification (16 cycles) was performed on the cDNA using a universal primer and a barcoded primer. Final size selection was performed on 3% gel cassette on a Pippin HT between 126 pb and 169 pb. Sequencing (single read 50 nucleotide) was performed using a NovaSeq 6000 (Illumina, Evry, France) with SBS (Sequence By Synthesis) technique. A total of over 449 million reads (sequences after Illumina Purity Filter) were obtained, with a number of reads per library ranging between 24 and 52 million. All reads were deposited into NCBI Sequence Read Archive under accession number SUB9869985. Reads were trimmed using cut adapt to remove the adapter sequence GCCTTGGCACCCGAGAATTCCA and the random primers. Reads were subsequently aligned onto the trout genome (NCBI RefSeq assembly accession GCF_013265735.2).

### 2.7. c-miRNA Annotation, Quantification and Differential Expression Analysis

The trout miRNAome annotation was established using Prost! [40], which was ran on the trout reference genome (NCBI RefSeq assembly accession GCF_013265735.2). Prost! was ran using the trout c-miRNA annotation, as previously established [31], for all the circulating fluids sequencing samples.

When Prost! identified multiple potential annotations for a given sequence, counts for this sequence were distributed relatively to the abundance of single annotated sequences of the same c-miRNA. If a c-miRNA was found only in multiple annotation, counts were distributed evenly between annotations. Counts in samples from the same tissue were averaged, and c-miRNAs for which normalized abundance was greater than 10 RPM were considered expressed in the fluid. Differential-expression analyses were performed using DESeq2 [41] and raw counts from all fluid c-miRNA-expression data. For each differential-expression test, log fold changes were corrected using lfcShrink (type = “apeglm”), and p-values were corrected using an FDR method (correction of Benjamini-Hochberg) to account for multiple testing. To identify c-miRNAs differentially expressed between the different conditions, a model was built using all samples and considering all possible effects (~condition).

To investigate the possible tissue origin of c-miRNAs differentially expressed in blood plasma, we used small sequencing libraries of samples originating from a wide variety of tissues in precedent studies [42]. This analysis investigated a subset of 14 different organs from females (brain, pituitary, gills, liver, heart, muscle, stomach, intestine, spleen, head-kidney, trunk-kidney, skin, ovary, eggs).

### 2.8. Statistical Analysis

All statistical analyses were performed using R software (v3.5.2)/R Commander package. All data are expressed as mean (EE vs. BE) with standard deviations across biological replicates. Effects were considered statistically significant at *p* < 0.05. Analyses were carried out on untransformed data, since criteria for normality and homogeneity of variances were fulfilled (Shapiro–Wilk and Levene’s test, respectively). Values of mRNA level of all transcripts revealed by RT-qPCR were analysed by Welch’s *t*-tests. If the criteria (normality and homogeneity) were still not met, a non-parametric test was used for the analysis.

## 3. Results

### 3.1. Influence of EE on mRNA Expression Levels of Neuronal Activity Gene in Trout Brain

**Figure 2:**
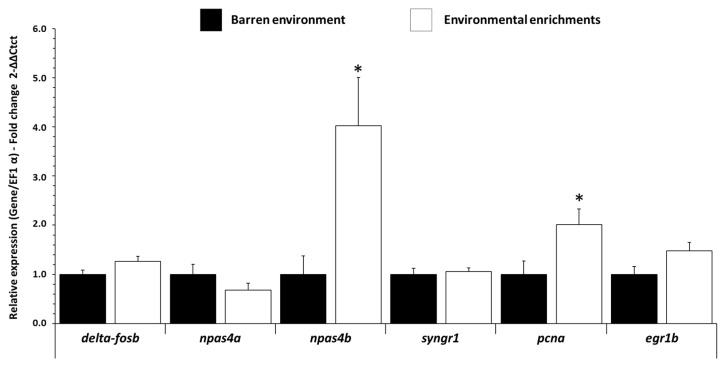
Neuronal activity related mRNA level in forebrain of trout raised in a barren environment (black) or environmental enrichments (white) for three months. Values are expressed as group mean ± SEM; fold change vs. barren environment for all genes; Welch’s t-test, Tukey post hoc; differences between treatments are represented by * (*p* < 0.05). Replicates (n = 8) correspond to different individual fish.

In the forebrain, *npas4b* (Neuronal PAS Domain Protein 4) and *pcna* (proliferating cell nuclear antigen), early markers of excitatory-inhibitory balance of neural circuits and markers of neurogenesis, respectively, were increased (*p ˂* 0.021 f = 2.98 and *p ˂* 0.031 f = 2.439, respectively) in EE fish compared to BE fish (Figure 2). Other genes (*delta-fosb, syngr1*, *egr1b*) were not significantly differentially detected in the forebrain and hypothalamus (Figure S1), and no mRNA-expression-levels variations were detected in the hindbrain for any of the genes studied (Figure S1).

### 3.2. Influence of EE on mRNA Expression Levels of Neurotrophic and Synaptogenesis Gene in Trout Brain

**Figure 3:**
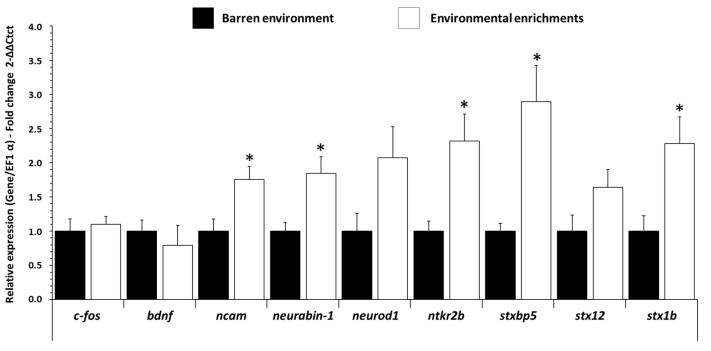
Neurotrophic and synaptogenesis factors related mRNA level in forebrain of trout raised in a barren environment (black) or environmental enrichments (white) for three months. Values are expressed as group mean ± SEM; fold change vs. barren environment for all genes; Welch’s *t*-test, Tukey post hoc; differences between treatments are represented by * (*p* < 0.05). Replicates (n = 8) correspond to different individual fish.

In the forebrain, *ncam* (Neural Cell Adhesion Molecule, marker of neural cell migration and adhesion), *neurabin-1* (marker of neurite formation), *ntrk2b* (neurotrophic tyrosine kinase, receptor, type 2, marker of neurons survival and differentiation), *stxbp5* (Syntaxin Binding Protein 5, marker of synaptogenesis), and *stx1b* (Syntaxin-1b, marker of synaptogenesis) were increased (*p ˂* 0.012 f = 2.948, *p ˂* 0.014 f = 3.024, *p ˂* 0.019 f = 3.127, *p ˂* 0.011 f = 3.474, *p ˂* 0.018 f = 2.84, respectively) in EE fish in comparison to BE fish (Figure 3). Other genes (*c-fos, bdnf, neurod1*, *stx12*) were not modified in the forebrain, and no mRNA-expression-levels changes were detected in the hypothalamus and hindbrain (Figure S2).

### 3.3. Influence of EE on mRNA Expression Levels of Plasticity Gene in Trout Brain

**Figure 4:**
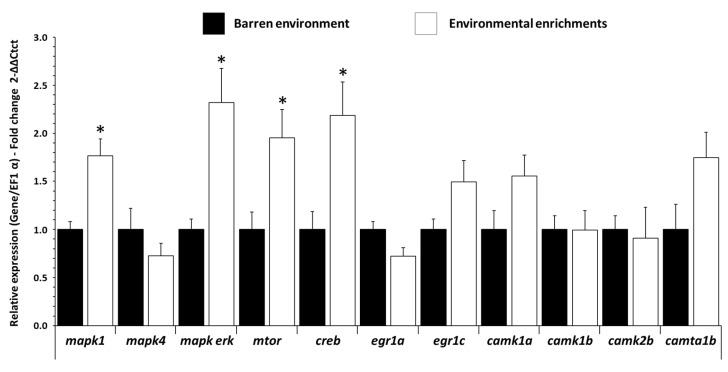
Plasticity factors related to mRNA level in forebrain of trout raised in a barren environment (black) or environmental enrichments (white) for three months. Values are expressed as group mean ± SEM; fold change vs. barren environment for all genes; Welch’s *t*-test, Tukey post hoc; differences between treatments are represented by * (*p* < 0.05). Replicates (n = 8) correspond to different individual fish.

In the forebrain, *mapk1* (Mitogen-activated protein kinase 1, marker of synaptic plasticity and cell proliferation), *mapk-erk* (marker of neurite formation), *mtor* (mammalian target of rapamycin, marker of plasticity, proliferation, growth and survival), and *creb* (C-AMP Response marker of synaptic plasticity and cell proliferation) were increased (*p ˂* 0.003 f = 3.973, *p ˂* 0.009 f = 3.552, *p ˂* 0.021 f = 2.744, *p ˂* 0.015 f = 2.997, respectively) in EE fish compared to BE fish (Figure 4). Other genes’ expressions (*mapk4, egr1a, egr1c*, *camk1a, camk1b, camk2b*, *camta1b*) were not modified in the forebrain, and no changes in mRNA levels were detected in the hypothalamus and hindbrain (Figure S3).

### 3.4. Influence of EE on mRNA Expression Levels of Neurotransmitters Pathways Gene in Trout Brain

**Figure 5:**
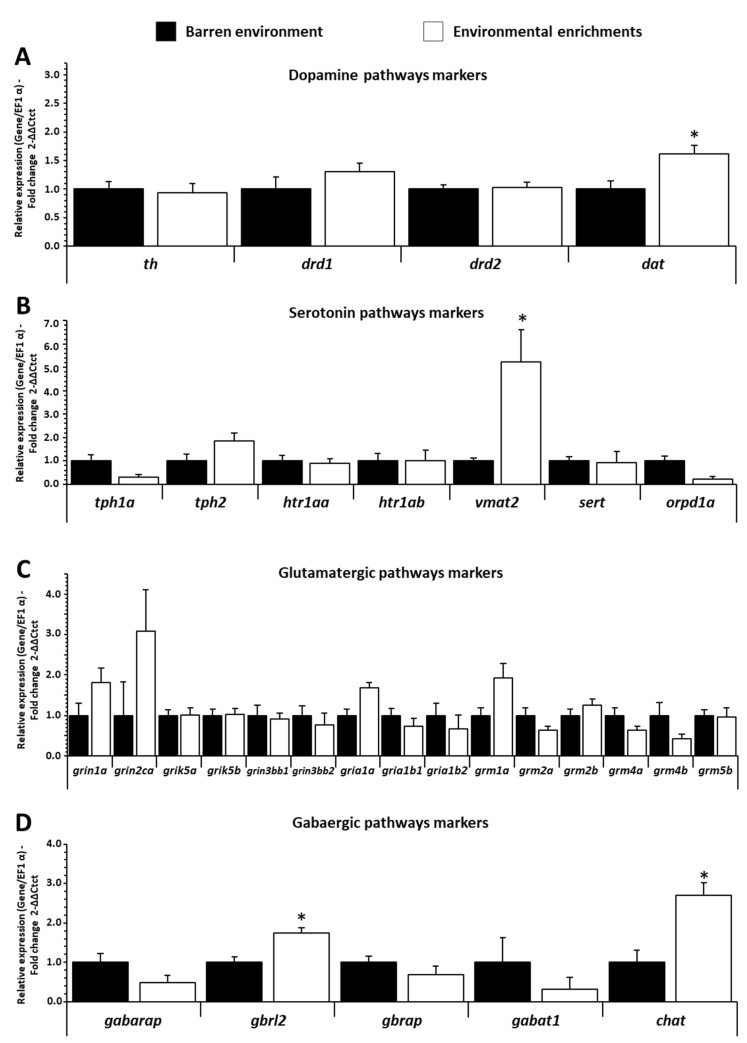
(**A**) Dopamine, (**B**) serotonin, (**C**) glutamatergic, and (**D**) gabaergic pathways related mRNA level in forebrain of trout raised in a barren environment (black) or environmental enrichments (white) for three months. Values are expressed as group mean ± SEM; fold change vs. barren environment for all genes; Welch’s *t*-test, Tukey post hoc; differences between treatments are represented by * (*p* < 0.05). Replicates (n = 8) correspond to different individual fish.

For dopamine pathways, only *dat* (dopamine carrier) was increased (*p ˂* 0.013 f = 2.907) in the forebrain of EE fish compared to BE fish (Figure 5A). In contrast, *th* (tyrosine hydroxylase enzyme)*, drd1*, and *drd2* (dopaminergic receptor) were not differentially expressed in the forebrain, and no mRNA-expression-levels changes were detected in the hypothalamus and hindbrain of trout (Figure S4A).

For serotonin pathways (Figure 5B), only mRNA *vmat2* (vesicular monoamine transporter 2) in the forebrain was increased (*p ˂* 0.021 f = 3.064) for EE fish compared to BE. *tph1a* and *tph2* (precursor isoenzyme of serotonin), *htr1aa* and *htr1ab* (serotonin receptor), and *sert* (serotonin carrier) were not regulated in the forebrain, and no mRNA-expression-levels changes were detected in the hypothalamus and hindbrain of trout (Figure S4B).

For glutamatergic pathways (Figure 5C), for all selected genes studied, no genes (*grin1a, grin2ca, grik5a, grik5b, grin3bb1, grin3bb2, gria1a, gria1b1, gria1b2, grm1a, grim2a, grim2b, grm4a, grm4b*, *grim5b*) were regulated in the forebrain, hypothalamus, and hindbrain of trout (Figure S4C).

For gabaergic pathways (Figure 5D), *gbrl2* (Gamma-aminobutyric acid (GABA) receptor-associated protein-like 2) and *chat* (enzyme of GABA) expression in the forebrain was increased (*p ˂* 0.002 f = 3.915 and *p ˂* 0.006 f = 3.614, respectively) for EE fish compared to BE. The expressions of *gabarap*, *gbrap* (GABA receptor), and *gabaT1* (GABA carrier) were not different in the forebrain, and no changes were detected in the hypothalamus and hindbrain (Figure S4C).

### 3.5. Influence of EE on Selected c-miRNAs Abundance in Blood-Plasma Fluid in RT

**Figure 6:**
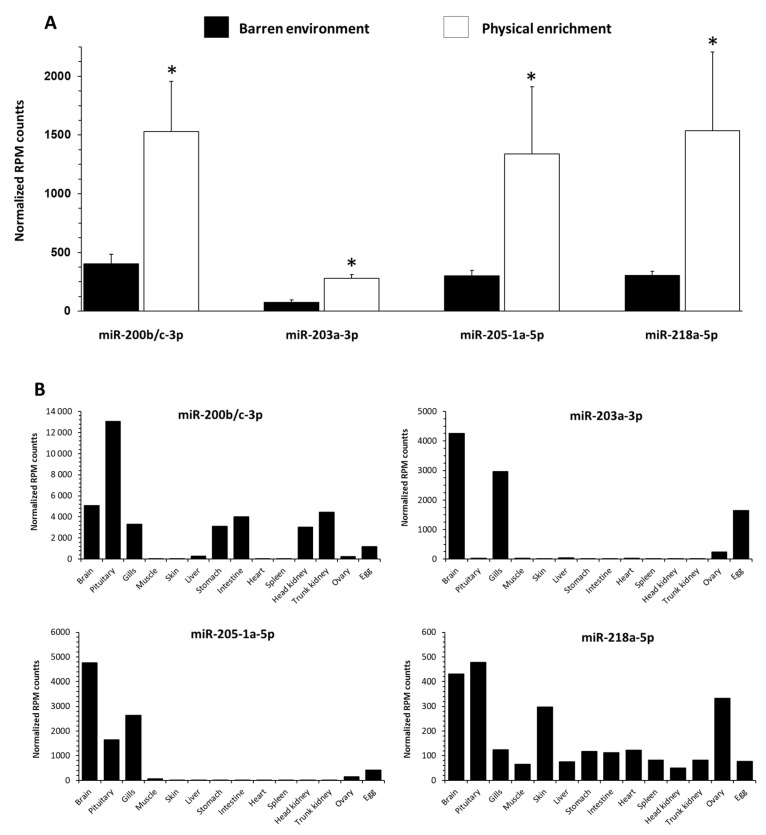
Abundance and tissue origin of c-miRNAs differentially expressed in blood plasma of fish raised in a barren environment (black) or environmental enrichments (white) for three months. (**A**) Abundance of c-miRNAs differentially expressed (*p* ˂ 0.05, see asterisk) in blood plasma. Values are expressed as group mean ± SEM; differential expression analyses were performed using DESeq2 and raw counts from all fluid c-miRNA expression data. For each differential expression test, log fold changes were corrected using lfcShrink (type = “apeglm”), and p-values were corrected using an FDR method (correction of Benjamini-Hochberg) to account for multiple testing. Replicates (n = 5 for EE treatment or 6 for BE treatment) correspond to different individual fish. (**B**) Abundance of c-miRNAs differentially expressed in blood plasma in a panel of 14 tissues. Values are from small sequencing libraries of precedent studies [31,42].

Among the 354 known annotated trout c-miRNAs [31], 203 were detected on average above a threshold of 10 reads per million reads (RPM). A significant upregulation of four c-miRNAs was observed in trout reared with EE: miR-203a-3p levels were increased with a log two-fold change of 1.62 (*p ˂* 0.01), miR-205-1a-5p with a log two-fold change of 1.24 (*p ˂* 0.01), miR-218a-5p with a log two-fold change of 1.37 (*p ˂* 0.01), and miR-200b/c-3p with a log two-fold change of 1.38 (*p ˂* 0.02) (Figure 6A).

The potential tissue origin of these candidate c-miRNAs was analyzed in a panel of tissues to shed light on their possible origin of expression (Figure 6B). MiR-218a-5p and miR-200b/c-3p were expressed in a wide variety of tissues with a maximum of expression in the brain (including the pituitary gland), whereas miR-203a-3p and miR-205-1a-5p appeared as predominant in the brain and gills.

## 4. Discussion

In this study, we observed that trout reared in an enriched environment displayed a differential expression of several genes and pathways related to plasticity, neurogenesis, and neuronal function mainly in the forebrain. The present study also demonstrated a c-miRNA signature in the plasma for fish reared in EE, suggesting their physiologically relevance as biomarker candidates for the effects of EE in trout.

### 4.1. EE Affects Cerebral Function in Telencephalon of Trout

In fish, numerous studies reported the impact of EE on brain development, gene expression, and behavior and learning abilities [7,9,17,43,44,45]. While these studies indicated that EE affects gene-expression patterns, it remains challenging to interpret how these neurobiological differences may be linked to behavior because very few studies have compared genes related to pathways linked to brain activity and behavior in specific brain areas between EE vs. BE, and none have compared them in trout. In the present study, all selected brain markers differentially expressed between the fish reared in BE vs. EE were mainly located in the forebrain. In vertebrates, cognitive processing is mainly under forebrain control, and, more specifically, the cerebral cortex [46]. In contrast to mammals, teleost fish do not possess a cerebral cortex. Furthermore, fish telencephalic areas (containing the amygdala and hippocampus) have been found to be functionally equivalent to mammalian forebrain regions [47,48], and fish are capable of displaying complex behaviors including decision-making and associative learning as well as the cognitive regulation of emotion, which are under forebrain regulation [49]. We previously showed that fish environmental perception, which is a cognitive process leading to various emotional states (anxiety, fearfulness, etc.) [15], is influenced by a complex and stimulating environment, [14]. Our new results suggest that, as in mammals, these cognitive EE effects are mainly under forebrain control. Environmental stimulations likely enhanced functional adaptation of this brain area via synaptic plasticity, neurogenesis, and neuronal activation, possibly causing the low-anxiety profile observed in fish raised in an environmental enrichment [14].

Among functional adaptation, it is well known that external stimulations such as those provided by the environment have a variety of effects on brain plasticity at levels ranging from the molecular and cellular to the behavioral [50]. In particular, EE induces many structural and functional changes in the brain, especially neuroplasticity, and it is noteworthy that these changes result in further beneficial effects at behavioral levels (for review, see [51]). Neuroplasticity induces cognitive and emotional changes characterized by several observed behavioral modifications: improved spatial and non-spatial learning and memory, novel object discrimination, enhanced spatial searching strategies, and decreased anxiety-like behaviors [51]. In fish, environmental complexity can lead to neural developmental and plastic modifications; and if understimulated, fish (e.g., *G. morhua*) develop smaller brains [52].

In the present study, we found that some brain plasticity markers were upregulated in the forebrain of enriched fish. Among the molecular brain factors revealing brain plasticity, *mtor* took center stage in the molecular studies of neuronal plasticity in mammals [53]. In particular, *mtor* allows an increase in the rate of translation in response to external stimuli, and *mtor* has been repeatedly shown to participate in neuronal development and the proper functioning of mature neurons enhancing neuronal survival, differentiation, and morphogenesis [54]. In mice, blocking the activity of the mTOR protein is known to trigger reduced social behavior, attention, and spatial learning [55]. In fish, mTOR is particularly important in the consolidation of long-term memory [56]. In the same way, MAPK/ERK is a protein kinase activated by neurotrophic factors involved in synapse formation and plasticity, which has a dual role in the establishment of functional synaptic connections and their short-term plasticity [57]. It is also well established that the MAPK/ERK kinase signaling cascade plays critical roles in brain development, learning and memory [58]. In addition to its role in plasticity and behavior, the ERK pathway, which can be revealed by CREB expression, is crucial in the cellular and molecular mechanisms underlying the behavioral effects related to brain plasticity [59]. All these studies in mammals suggest that molecular signaling pathways related to brain plasticity could also play a prominent role in the modification of the cognitive processes due to EE in trout. However, a more in-depth analysis, particularly at the protein level, will support this conclusion.

In addition to markers of neuroplasticity, markers of neuronal activation highlight the influence of EE on the adaptive capacity of the cerebral activation underlying the cognitive processes. Among them, the transcription factor neuronal PAS domain-containing protein 4 (*npas4*) has been suggested as a candidate gene in the cortex of mammals (amygdala and hippocampus), regulating anxiety or depression [60], cognition [61], and social behavior [62]. Here, we found a decrease in the expression of *npas4b* in the hypothalamus of EE fish. This could indicate that EE significantly stimulates the neuronal circuit of fish brain, likely explaining the decrease in the anxiety-like behaviors we previously observed in EE fish and their willingness to explore a novel surrounding perceived as less threatening than by BE fish [14].

Activation of neuronal circuits can be mapped through visualization of immediate early neurotrophic genes such as *bdnf, c-fos*, and *neurod1*. In the few fish studies, lower expression of *bdnf* [43] and upregulation of *neurod1* [17] in the forebrain subregions associated with cognitive functions were measured in enriched salmon. However, we did not observe any change in the expression of these two genes in EE fish. The duration of the enriched period before brain-gene analyses was relatively long (i.e., three months), and transcripts were not immediately quantified after a behavioral test, which could explain that the visualization of immediate early genes *bdnf* and *neurod1* were not different between groups. Nevertheless, with nine selected established markers, our study showed an important upregulation for five of them in EE fish. *ncam*, which regulates neuronal development by modulating cell adhesion and signal transduction, is known to play an important role in learning skills in mice, by enhancing stabilization of synaptic contacts [63]. This marker is also an important contributing factor to the pathophysiology of depression in mammals. Constitutive deficiency of *ncam* in mice resulted in impaired memory and led to an increased immobility in the tail-suspension test [64], suggesting a depression-like behavioral phenotypes [65]. In accordance with these observations in mammals, a more in-depth analysis at the protein level could suggest that *ncam* activity may be associated with lower anxiety-related behaviors and higher exploration for fish reared in EE. There is also some evidence indicating a possible role for the *ntrk2b* receptor and its ligand in learning and memory [66]. Neurotrophins, including the NTRK receptor, are involved in the alteration of hippocampal function and behavior in mammals as a susceptibility gene in neuropsychiatric disorders [67]. Overall, upregulation of neural plasticity, neurogenesis, and neuronal activation markers in the forebrain of fish reared in a complex and stimulating environment could contribute, at least in part, to enabling the behavioral plasticity and the positive emotional state observed in our previous experiment [14], as shown by a lower anxiety level and a higher motivation to explore in these fish.

Regarding neurotransmitters, there are critical neuromodulators known to play a fundamental role in a multitude of cognitive processes, including reward processing, learning, decision-making, motivation to engage in goal-orientated behaviors (e.g., eating and drinking), or positive affects [68,69]. In fish, the central neurotransmitter system also influences behavior [70] and cognitive processes [71], including neural plasticity associated with learning and memory [72,73]. For example, the serotonergic system influences behavior [70] and cognitive processes [71], including the neural plasticity associated with learning and memory [73]. EE also increases dopaminergic and serotonergic activities in fish telencephalon, in link with enhanced cognition and exploratory behavior [74]. Overall in fish, it is well known that the barren environment damaged these cognitive functions (spatial-learning ability) in striped knifejaw (*Oplegnathus fasciatus*) [75], rainbow trout (*Oncorhynchus mykiss*) [76], zebrafish (*Danio rerio*) [77], guppies (*Poecilia reticulata*) [78], and gilthead seabream (*Sparus aurata*) [74]. In the present experiment, despite the evidence of the impact of EE in the regulation of neurotransmitters in fish, related genes remain mainly undifferentiated between the two treatments. Of the 4 genes involved in the dopamine system, only *dat* was upregulated in the forebrain of EE fish; for the serotonin system, only *vmat2* was upregulated in the forebrain of the 6 genes; 1 gene (*gria1b1*) was under-regulated in the hypothalamus of the 15 targeted genes involved in the glutaminergic system, and 2 (of the 5) genes involved in the GABAergic pathway were upregulated in the forebrain. Although few in number, the upregulated genes involved in the regulation of the central neurotransmitter system were localized in the forebrain of EE fish, supporting the role played by this brain area in the underlying effects of environmental enrichment on fish behavioral plasticity.

### 4.2. EE Influences Circulating miRNAs Profiles of Plasma in Trout

Besides showing that the molecular events associated with EE-induced behavioral plasticity and cognitive processes are associated with changes in gene expression, we hypothesized that this influence (and associated molecular changes) might induce changes in c-miRNAs in blood plasma. While roles for c-miRNAs in non-pathological contexts have received little attention, and data in non-human species remain scarce and mostly related to pathologies, we investigated whether changes in c-miRNAs could be linked to the behavioral plasticity observed in our enriched fish [14]. We found that four c-miRNAs, miR-200b/c-3p, miR-203-3p, miR-205-1a-5p, and miR-218a-5p, were upregulated in EE fish that could be linked to the modified cognitive perception of the environment, as shown by the fewer anxiety-like behaviors in these fish. The link between these four c-miRNAs and behavior is difficult to establish, given the lack of research on this subject in fish, especially for c-miRNA. However, a link between miR-200-3p and serotoninergic changes was established in rats [79]. More specifically related to behavior, miR-200-3p seemed to influence agonistic behavior in the Chinese mitten crab (*E. sinensis*) by mediating the serotonin- and dopamine-regulation mechanisms [28]. MiR-205-3p was significantly dysregulated in mice with tauopathy, a neurodegenerative disease that includes deficits in cognition, behavior, and movement [80]. In addition, miR-218-5p was one of the most significantly induced c-miRNAs in the prefrontal cortex of rats under chronic stress and plays a functional role in depression- and anxiety-like behaviors [81]. Moreover, a link between miR-218-5p and dopaminergic neurons in rats is established [82]. In our study, the upregulation of serotonin (*vmat2*) and dopamine transporters (*dat*) playing a role in the re-uptake of these monoamines could suggest changes in the activity of these neurons, thus being able to regulate cognitive functions and behaviors in fish reared in EE. These four c-miRNAs are either specific to certain tissues—this is the case of miR-205-1a-5p and miR-203a-3p, which mainly originate from the brain and gills (to a lesser extent)—or present in a majority of tissues, but with a maximum expression in the brain (miR-200b/c-3p and miR-218a-5p). The high presence of these c-miRNAs in the brain suggests that the brain could contribute to differential c-miRNA levels in blood plasma.

We, thus, provide evidence that rearing fish with EE results in the behavioral plasticity associated with brain genes’ modulations and changes in the level of certain c-miRNAs in blood plasma. Despite a lack of clear understanding of the biological roles of c-miRNAs, these promising results highlight the potential of c-miRNAs as physiologically relevant biomarkers in fish cognitive processes and behavior. Moreover, these results pave the way for the use of c-miRNAs for non-invasive phenotyping of behavioral plasticity in fish.

Finally, the results of this study illustrate that the living conditions of trout (EE vs. BE) are associated with important modifications (markers of plasticity, neurogenesis, cerebral activity) in brain regions that are key for their adaptation. Thus, these modifications illustrate that environmental conditions can have major consequences on the functioning of their brain and, potentially, on their welfare and adaptation to the proposed conditions.

## 5. Conclusions

Our previously published study [14] revealed important behavioral plasticity in trout, probably linked to their perception of a less-threatening environment when reared with enrichments. Perception is a cognitive process, causing different emotional states, which are mainly expressed here by lower fearfulness towards novelty and fewer anxiety-like behaviors. As far as we are aware, by complementing this previous work, we demonstrated for the first time in a teleost fish that complexifying the environment through the addition of physical structures enhances neuroplasticity, which promotes neuro-synaptogenesis and neural activation in the fish forebrain, which are important for cognitive processes, providing a neuronal foundation for the behavioral differences observed between BE and EE. This study also demonstrated a c-miRNA signature in plasma for fish reared in EE, suggesting their physiological relevance as biomarker candidates of cognition-related behavioral plasticity in trout. 

## Figures and Tables

**Table 1:** In batch 1: mean fish weight, tank size, number of individuals, number, type and size of structures, and estimated floor covering at each stage.

Days Post-Fertilization	Average Fish Weight at the Beginning of Each Period	Tank size	Number tank Replicates and Number of Individuals	Number (n) and Type of Structures Used in the Enriched Group			Floor Covering
				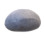	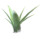	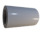	
53–210	0.12 g	100 L (65 × 50 × 30 cm) Covered tanks	3 tanks/treatment 100 individuals each	n = 1 (10 × 5 × 5 cm)	n = 2 (24 × 15 × 11 cm)	n = 2 (9 × 8 × 8 cm)	~60%
210–238	18.6 g	72 L (55 × 45 × 29 cm) Uncovered tanks	1 tank/treatment 30 individuals each	n = 1 (10 × 5 × 5 cm)	n = 2 (24 × 15 × 11 cm)	n = 2 (9 × 8 × 8 cm)	~70%

**Table 2:** In batch 2: mean fish weight, tank size, number of individuals, number, type and size of structures, and estimated floor covering at each stage.

Days Post-Fertilization	Average Fish Weight at the Beginning of Each Period	Tank Size	Number tank Replicates and Number of Individuals	Number (n) and Type of Structures Used in the Enriched Group			Floor Covering
				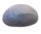	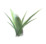	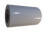	
99–136	2.83 g	72 L (55 × 45 × 29 cm) Uncovered tanks	2 tanks/treatment 100 individuals each	n = 1 (10 × 5 × 5 cm)	n = 2 (24 × 15 × 11 cm)	n = 2 (9 × 8 × 8 cm)	~70%
137–189	11 g		3 tanks/treatment 30 individuals each			n = 2 (9 × 8 × 8 cm)	~70%

**Table 3:** Nucleotide sequences of the PCR primers used to evaluate mRNA abundance of genes by RT-PCR (quantitative PCR).

Transcript	Forward Primer	Reverse Primer	Accession Number
**Reference**			
*eef1a1*	TCCTCTTGGTCGTTTCGCTG	ACCCGAGGGACATCCTGTG	AF498320
*Neuronal activity*			
*delta-fosb*	TGCAGCCAACTCTCATCTCG	GAGGAATAACTGGGCCCTGG	XM_021587380.1
*npas4a*	GAGATGGTGTTGCAGGTGGA	TAGTTGTGGCAGCTGATGGG	XM_021618560.2
*npas4b*	AGCGAGAGAGGGAGGACATT	GTGGGTGGGGTTATTCTGGG	XM_036967710.1
*syngr1*	CTTCCCACAGATCAGCTCCG	CAGGAAACAGAACCCCACGA	XP_036805055.1
*pcna*	GTGGACAAGGAGGAGGAAGC	ACTGTCTTGGAGAGGGGTGT	XM_036936092.1
*egr1b*	CCCAACATGTCTCTGCCCAT	GCTCTGACACTGGAAAGGCT	XM_021617535.1
*Neurotrophic factor*			
*c-fos*	AACAGACTCTCCATGGCAGT	TGCTGATGTGATGACGGTGG	XM_021611391.1
*bdnf*	GCTGCCGTGGAATAGACAAG	TCCTTATAAACCGCCAGCCA	GU108576.1
*ncam*	GCTAACGTCACCAAAGCCAA	GGCAGCAGTACAGTTGTAGC	XM_021582629.1
*neurabin-1*	AGGAGAGAGAGGAGACAGCA	TTCCTCCGCCTGTTTCTCAT	XM_021572886.1
*neurod1*	AACCATGAGTAAGGACGGCG	TTCTCCCGACCCTCCTTCTT	XM_021608264.2
*ntkr2b*	ACTATCCTGGAGCTGCTGGA	CTGTTCGTGGAGCTCTTGGT	XM_021602993.2
*stxbp5*	GTCCTCCAAGTCACACCCTG	TCAGATCCCACAACACCACG	XP_021441002.2
*stx12*	ACAACTTCCAGGCCGTACAG	CAGAGGCACCATCTTCAGCA	XM_036970689.1
*stx1b*	TGACCGAGTACAACACCACG	GCATATCCTCCAGCTCCTCG	NP_001117929.1
*Plasticity/synaptogenesis factor*			
*mapk1*	CCTGCTCATCAACACCACCT	AGCCACGTACTCTGTCAGGA	XM_036937960.1
*mapk4*	GGCAGGCTCTAAACCCTTGT	AGAGAGGAGTGGGAGTGGTC	XM_021620877.2
*mapk-erk*	AGTCCATCTCCACGACCATC	GAAAGCCTCCAGACGTTTCC	NM_001124424.1
*mtor*	CCAAGGACTTCGCTCACAAG	GCTCCTTGATGTCTTGCTGG	XM_021615845.1
*creb*	CAGATTGACAGCTGCCCCTA	TGGTGTTCTGGTGTAGTGCT	MG310160.1
*egr1a*	CGATCACCTGACCACACACA	TCAGGTGGATCTTGGTGTGC	ENSOMYT00000041426.1
*egr1c*	CTCGTACCCCTCTCCCTCAA	AGATGGAGGCTACGGAGGAG	ENSOMYT00000036541.1
*camk1a*	AGAGGACGGGAATGGATGGA	CTAGCACCACCTCCGAGAAC	XM_036950122.1
*camk1b*	GTTGGCCCAGAAACCCTACA	AGAGCTTGGCGTCATTCTCA	NM_001124638.1
*camk2b*	GCCGCTGTGTCAAACTTTGT	GCTTCCCTCTCCAGCTTCTG	XM_036976944.1
*camta1b*	CTACTGCCCTGCCCATGAAA	GGGCAAGTCTCGAGCTTTA	XM_036947092.1
**Dopamine markers**			
*th*	ACGCTCTCTCAAGGTGTTCG	AAAGTACTCCAGCCCCTCCA	XM_021564247.1
*drd1*	GGAGGAGCTGCAGAAGAAGG	TTTCCAGTGACACATCGGCA	XM_021617454.1
*drd2*	CCTCCAGTCCACCACCAATT	CCACTCTCCCACCACCTCTA	NM_001124372.2
*dat*	CTACCTCAGCGTCGACTTCC	TAGCACACCAAACCCGACTC	XM_021592557.1
**Serotonin markers**			
*tph1a*	ACACCAGAGCCAGACACATG	TCATCTGAAGCTCCGAGGGA	XM_021598622.1
*tph1b*	AGCGTCCGTTTACAGTGAGG	GCCCACGATGTCCAGTTCAT	XM_021598622.1
*tph2*	AGCACCTCAAAGACCACGTC	ACTGGTCGAGCTCTGCAATC	MG015698
*5ht1aa*	CCCAACACTCCACAGTCCTC	ACCGAGCGTCTTTACCGTTT	XM_021622104
*5ht1ab*	GAGGACCAACGGGGACCCGA	AATCGCCGTGCTTGACCGCA	CCAF0100015582
*sert*	CCTGCTGCCCTACATGTTGA	GGGGCAGATGTGTTTCCAGA	M_021582096.1
**Glutamatergic markers**			
*grin1a*	AACAAGCGAGGACCTAAGGC	CTGGCGGAGAGGATGATGAC	XM_021602512.2
*grin2ca*	ACCCTCTGCCTTTCTTGAGC	CACAGGGCTGCAGTACTCAA	XM_036938636.1
*grik5a*	GCAGATCAGGGTCCAGTCAC	AGTCAAAATACCCTCCCGCG	XM_036969398.1
*grik5b*	TGAAGAGGAGGTGGTGGGAA	GATGATGAGGCCGCAGATCA	XM_036952756.1
*grin3bb1*	CTACTTCAGTGAGCGTGCCA	TACTCGAAGCGCATGTCCTC	XM_021614590.2
*grin3bb2*	GGATCCAGAATAGGCCTGCC	GAACACCCTCTTCCCACAGG	XM_036977945.1
*gria1b1*	GCCTTTCAGAACCTCCGGAA	CTGGATGTCGATACCCTGGC	XM_036989628.1
*gria1b2*	GCGTATTGACATTTCCCGGC	CCTCAATCCGAACCTGCTGT	XR_005035026.1
*grm1a*	GCTGATCGAAAGTGTGGGGA	ATGTTGGGGAGCAGGAAAGG	XM_021600740.2
*grim2a*	TGCATCGCCACTTCAGCTAA	TGCGTGTGAAGAGGATGACC	XM_036950357.1
*grim2b*	GTGAGGGGAAGTGAGACAGC	GGGGTTCCGTGTGTTAGTGT	XM_021568769.2
*grm4a*	CCATTTCATCTGGGTGGGCT	CCTCTGATGGACTGGCGTTT	XM_021609206.2
*grm4b*	GTGCCAGAGACCTTCAACGA	GACTGCGAGGTCCCAAAGAA	XM_036988394.1
*grim5b*	GGGCATCCTGTTTGACGAGA	TGTCCCAGCTCCCTACGTTA	XM_036961649.1
**Gabaergic markers**			
*gabarap*	CAGATGCACTTTCCCTCCCC	TCAACCGAAATCCCCATCTCG	NM_001165091.1
*gbrl2*	AGAGAGAGATGGGGATGGCT	AGGATGCAAGGGTTGTGTCA	NM_001165109.1
*gbrap*	CTCACAGTGGGCCAGTTCTA	GAGGTGGGAGGAATGACGTT	NM_001165091.1
*gabat1*	GGTGATGGAGTTTTGGGAGC	TAAACCAGGACCCAAGCGAT	XM_021615563.1
*chat*	CATCATCGTGGCATGCAAGA	AGTTCTCCGCCATCTTCACT	XM_021581165

## Data Availability

All the data are available from the first author and can be delivered if required.

## Supplementary Materials

The following supporting information can be downloaded at: https://www.mdpi.com/article/10.3390/biology11081093/s1, Figure S1: Neuronal activity related mRNA level of transcripts in hypothalamus and hindbrain of RT raised in a barren environment (black) or environmental enrichment (white) during three months; Figure S2: Neurotrophic and synaptogenesis factors related mRNA level of transcripts in hypothalamus and hindbrain of RT raised in a barren environment (black) or environmental enrichment (white) during three months; Figure S3: Plasticity factors related mRNA level of transcripts in hypothalamus and hindbrain of RT raised in a barren environment (black) or environmental enrichment (white) during three months; Figure S4: Dopamine, serotonin, glutamatergic and gabaergic pathways related mRNA level of transcripts in hypothalamus and hindbrain of RT raised in a barren environment (black) or environmental enrichment (white) during three months.
