# Supplementary material for: Physical Enrichment Triggers Brain Plasticity and Influences Blood Plasma Circulating miRNA in Rainbow Trout (Oncorhynchus mykiss)
Source: Biology (Basel). 2022 Jul 22;11(8):1093. doi: 10.3390/biology11081093 (PMC9394377; doi:10.3390/biology11081093)
Supplement: Supplementary file 1 [file biology-11-01093-s001.zip › biology-1792548-supplementary.pdf]

SUPPLEMENTAL DATA AND FIGURES

Physical enrichment triggers brain plasticity in rainbow trout (*Oncorhynchus mykiss*) and influence on blood plasma circulating miRNA

Emilie Cardona[1,†], Valentin Brunet[2,†], Elodie Baranek[1], Léo Milhade[3], Sandrine Skiba-Cassy[1], Julien Bobe[2], Ludovic Calandreau[4], Jérôme Roy[1,*,†] and Violaine Colson[2,*,†]

[1]INRAE, Université de Pau et des Pays de l'Adour, E2S UPPA, UMR1419 Nutrition Metabolism and Aquaculture, Aquapôle, F-64310 Saint-Pée-sur-Nivelle, France; emilie.cardona@inrae.fr (E.C.); elodie.baranek@inrae.fr (E.B.); sandrine.skiba@inrae.fr (S.SC.) ; jerome.roy@inrae.fr (J.R.)
[2]INRAE, UR 1037, LPGP Fish Physiology and Genomics, Campus de Beaulieu, F-35042, Rennes, France; valentin.brunet@inrae.fr (V.B.); julien.bobe@inrae.fr (J.B.); violaine.colson@inrae.fr (V.C.)
[3]IRISA, INRIA, CNRS, Université de Rennes 1, F-35000, Rennes, France; leo.milhade@irisa.fr (L.M.)
[4]CNRS, IFCE, INRAE, Université de Tours, PRC, 37380 Nouzilly, France; ludovic.calandreau@inrae.fr (L.C.)
* Correspondence: violaine.colson@inrae.fr / jerome.roy@inrae.fr
† These authors contributed equally to this work.

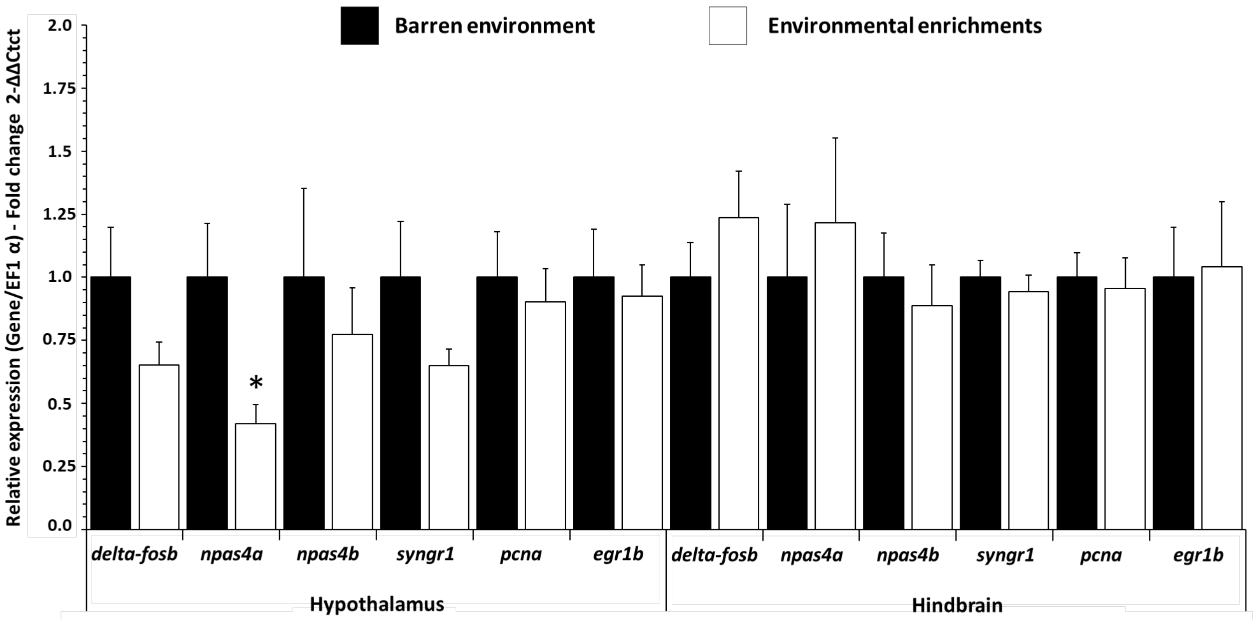

Figure S1. Neuronal activity related mRNA level of transcripts in hypothalamus and hindbrain of RT raised in a barren environment (black) or environmental enrichment (white) during three months. Values are expressed as group mean ± SEM; fold change vs barren environment for all genes; welch's *t*-test, Tukey post hoc; Differences between treatments are represented by *(P < .05). Replicates (n= 8) correspond to different individual fish.

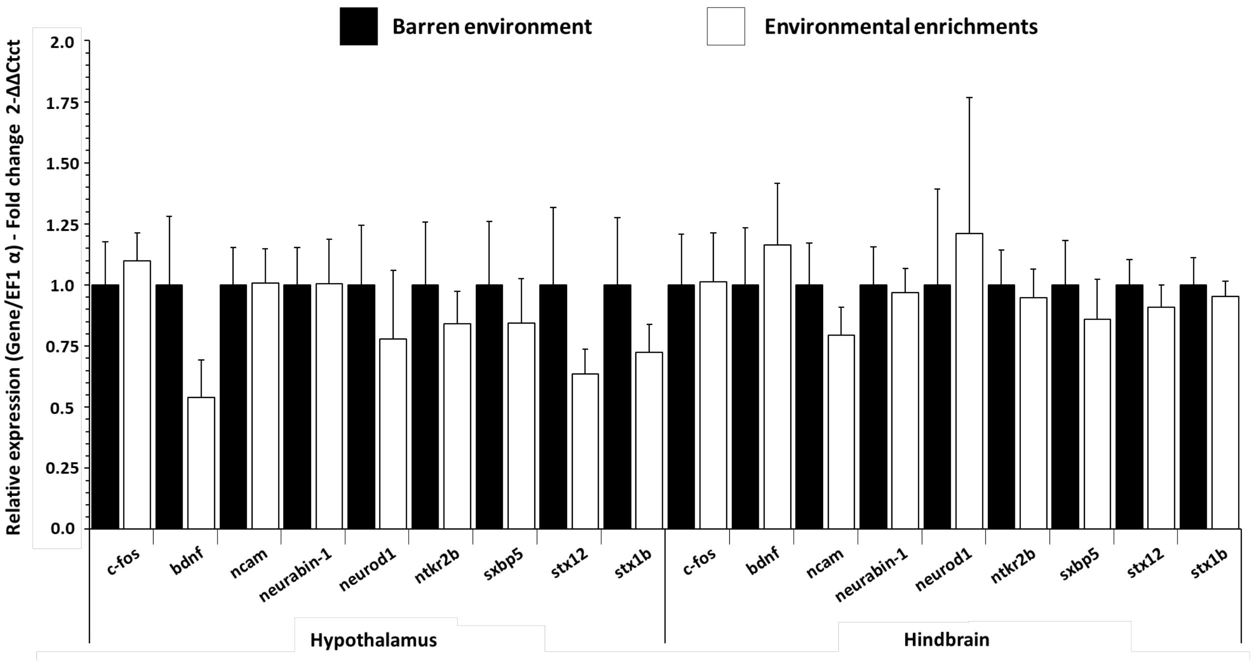

Figure S2. Neurotrophic and synaptogenesis factors related mRNA level of transcripts in hypothalamus and hindbrain of RT raised in a barren environment (black) or environmental enrichment (white) during three months. Values are expressed as group mean ± SEM; fold change vs barren environment for all genes; welch's *t*-test, Tukey post hoc; Differences between treatments are represented by *(P < .05). Replicates (n= 8) correspond to different individual fish.

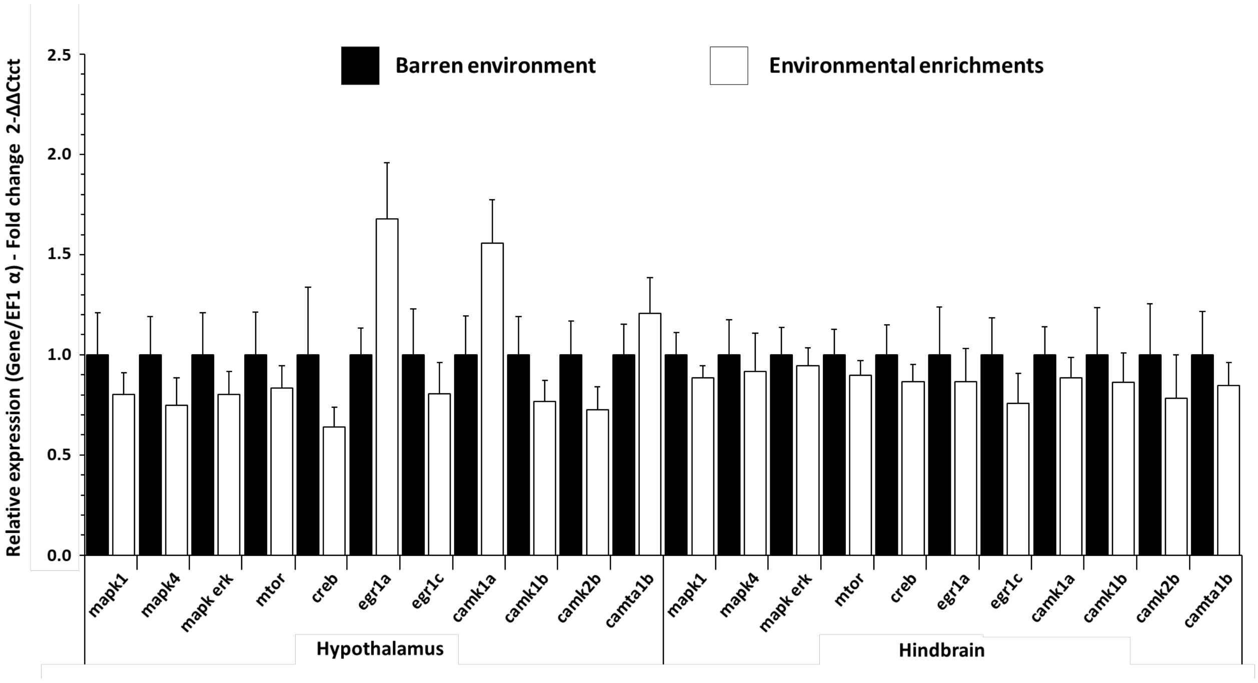

Figure S3. **Plasticity factors related mRNA level of transcripts in hypothalamus and hindbrain of RT raised in a barren environment (black) or environmental enrichment (white) during three months.** Values are expressed as group mean ± SEM; fold change vs barren environment for all genes; welch's *t*-test, Tukey post hoc; Differences between treatments are represented by *(P < .05). Replicates (n= 8) correspond to different individual fish.

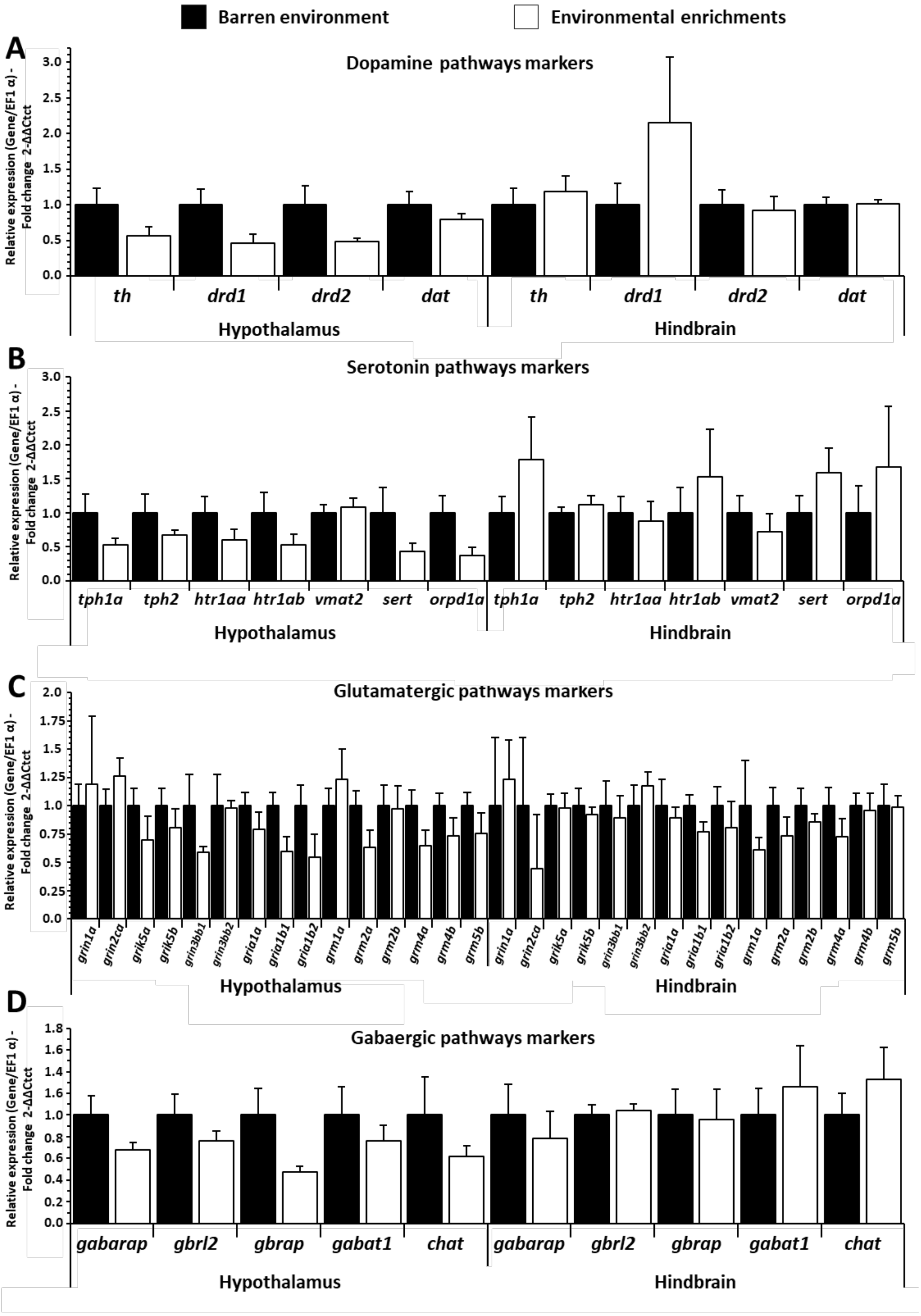

Figure S4. Dopamine, serotonin, glutamatergic and gabaergic pathways related mRNA level of transcripts in hypothalamus and hindbrain of RT raised in a barren environment (black) or environmental enrichment (white) during three months. Values are expressed as group mean ± SEM; fold change vs barren environment for all genes; welch's *t*-test, Tukey post hoc; Differences between treatments are represented by *(P < .05). Replicates (N= 8) correspond to different individual fish.
